# Commentary on picosecond Nd:YAG laser therapy for pigmentation due to lichen planus pigmentosus in a patient with skin of color

**DOI:** 10.1016/j.jdcr.2023.08.027

**Published:** 2023-09-01

**Authors:** Carlos G. Wambier

**Affiliations:** Department of Dermatology, Warren Alpert Medical School of Brown University, Providence, Rhode Island

**Keywords:** antiandrogens, FFA, fluence range, frontal fibrosing alopecia, isotretinoin, JAK inhibitors, JAKi, laser, lichen planus pigmentosus, lichen planus, LPP, Nd:YAG, neodymium-doped yttrium aluminum garnet laser, passes, pulses, ruxolitinib, sunscreens, titanium sapphire laser

*To the Editor:*

I read with interest the work by Belzer et al^1^ reporting partial improvement of postinflammatory pigmentation (PIH) in a patient with clinical diagnosis of lichen planus pigmentosus (LPP) who was submitted to a series of 10 treatments of picosecond Q-switched Nd:YAG laser 1064 nm with the Zoom handpiece at 3- to 4-mm spot size (Picoway, Candela) with uncertain fluence range (minimal “mJ” energy parameters provided), followed by 4 treatments with titanium sapphire crystal 785-nm handpiece at 3 to 4 mm with additional improvement (uncertain fluence range). This letter aims to confirm the fluence range used and to point out terminology minutia for readership and future reference.

Assuming the fluence range provided was “2.0 to 2.4 J/cm^2^” (high-fluence for picosecond 1064 nm) and 459 to 1212 were the total number of pulses (not “passes”), because that number of “passes” would likely be an extremely long session. I would comment that when treating melanophages/PIH vs dermal nevus, I avoid fluence ranges known to cause PIH by disrupting the basal layer, such as high-fluence 1064 nm, or utilizing wavelengths that cause epidermal frosting (532 nm or 785 nm). The authors could have described the “end point” aimed or achieved during the session with each parameter.

I have been treating LPP for >10 years using mild settings, such as those for melasma and PIH, a method coined in literature as “laser toning,” which consists of 1064 nm low-fluence settings with a wide spot size, such as the photoacoustic twin pulse mode (8 mm) 2.8 J/cm^2^ in conventional nanosecond Q-switched lasers,^2^ or “picotoning” (7 mm) 1 J/cm^2^ using PicoWay Zoom handpiece. In some patients with extreme dermal melanin deposits, severe pain may be elicited, such as a tattoo removal session, and dermal frosting (instant lightening of deep gray/blue pigment) can sometimes be noted, or just mild erythema and edema immediately after the session. When the photoacoustic feedback is too intense, lowering the parameters in the first session can avoid PIH. In my patients with frontal fibrosing alopecia, I combine antiandrogens (such as dutasteride) with low-dose isotretinoin, which was found to improve LPP.^3^

Although marketing teams in the picosecond laser industry have attributed the Q-switched terminology to refer to the “old” nanosecond devices, picosecond lasers are also Q-switched.^4^ When discussing previous literature, it is best to spell “nanosecond” or “picosecond” or specify the approximate duration of pulse (5 ns, 500 ps, etc). The authors have adopted a new term “Both Q-second and picosecond,” which emphasizes how the marketing was efficacious in imprinting Q-switching to “nano.”

Because LPP is an orphan disease, I congratulate the authors for publishing the case report. I was particularly impressed by the improvement in the perioral area at 1064 nm.

Through the looking-glass with the awareness by transcriptomic analysis that lichen planus is an interferon-driven disease and previous positive results of an open-label study,^5^ we hope to see reports of the employment of topical Janus Kinase inhibitors in combination with low-fluence Q-switched 1064-nm lasers (picosecond or nanosecond) and tinted zinc-based sunscreens in LPP (cases are currently being followed).

## Conflicts of interest

Dr Wambier has served as speaker for Cynosure; as an advisor for Vydence, Incyte, ChemistryRx, Young Pharmaceuticals, and Daniel Alain; and owner of Wambier Dermatologics, LLC.

## References

[1] Belzer A., Swallow M.A., Gowen M., Suozzi K.C. (2023). Picosecond neodymium-doped yttrium-aluminum-garnet laser therapy for pigmentation due to lichen planus pigmentosus in a patient with skin of color. JAAD Case Rep.

[2] Kim J.Y., Choi M., Nam C.H. (2016). Treatment of melasma with the photoacoustic twin pulse mode of low-fluence 1,064 nm Q-switched Nd:YAG laser. Ann Dermatol.

[3] Muthu S.K., Narang T., Saikia U.N., Kanwar A.J., Parsad D., Dogra S. (2016). Low-dose oral isotretinoin therapy in lichen planus pigmentosus: an open-label non-randomized prospective pilot study. Int J Dermatol.

[4] Ross V., Naseef G., Lin G. (1998). Comparison of responses of tattoos to picosecond and nanosecond Q-switched neodymium:YAG lasers. Arch Dermatol.

[5] Brumfiel C.M., Patel M.H., Severson K.J. (2022). Ruxolitinib cream in the treatment of cutaneous lichen planus: a prospective, open-label study. J Invest Dermatol.

